# Decrease in the Internal Cerebral Vein Pulsation With Improvement of Patent Ductus Arteriosus in Premature Infants at the Risk of Intraventricular Hemorrhage: Two Interesting Case Reports

**DOI:** 10.7759/cureus.65030

**Published:** 2024-07-21

**Authors:** Kenichi Tanaka, Shirou Matsumoto, Narumi Yoneda, Yusuke Hattori, Kimitoshi Nakamura

**Affiliations:** 1 Pediatrics, Kumamoto University, Kumamoto, JPN; 2 Neonatology, Kumamoto University Hospital, Kumamoto, JPN

**Keywords:** patent ductus arteriosus, brain natriuretic peptide, indomethacin, intraventricular hemorrhage, internal cerebral vein

## Abstract

Recently, augmenting the pulsation of the internal cerebral vein (ICV) has been reported to be a predictor of premature intraventricular hemorrhage (IVH); however, prophylaxis for IVH has not yet been established. Venous pulsation is a marker of central venous pressure elevation and may be improved after heart failure treatment. Herein, we report two cases of low-birth-weight infants (29 weeks and 31 weeks of gestational age), who exhibited improvements in ICV pulsation with relief of hemodynamically significant patent ductus arteriosus (hs-PDA) following indomethacin administration. ICV flow patterns were continuously flat early after birth. Thereafter, both patients demonstrated ICV pulsation augmentation with PDA progression and brain natriuretic peptide (BNP) elevation at 52 h and 39 h after birth (in infants born at 29 and 31 weeks of gestational age, respectively). After relieving PDA with indomethacin administration, both infants exhibited an improvement in ICV pulsation with decreased BNP levels. In both cases, ICV pulsation increased when PDA became hemodynamically significant with BNP elevation, and the pulsation improved by reduction in ductal flow with decreasing BNP when PDA was relieved by indomethacin administration. The association between hs-PDA and elevated ICV pulsation indicates that hs-PDA likely leads to heightened central venous pressure. Additionally, indomethacin treatment was effective in reducing the exacerbated ICV pulsation caused by heart failure due to hs-PDA. These cases suggest that treatment for heart failure might improve the augmented ICV pulsation, which is related to the development of premature IVH. However, further studies are needed to confirm this association.

## Introduction

Increased pulsation of the internal cerebral vein (ICV) is associated with intraventricular hemorrhage (IVH), a serious complication in premature infants [1]. The ICV forms a part of the deep cerebral venous system and drains most of the venous flow from the germinal matrix, which is a major IVH source [2,3]. We previously reported that augmented ICV pulsation is a predictor of premature IVH [4]; however, the method for IVH prevention after birth is not yet established.

Elevated right atrial pressure decreases venous wall distensibility, and changes in the right atrial pressure during the cardiac cycle are transmitted in the distal veins [5]. For example, the waveform of the portal blood flow is transiently reduced, interrupted, or retrograded, with the right atrial pressure being elevated [6]. Therefore, venous pulsation is a venous congestion marker that may be improved after treatment for heart failure.

We speculated that the ICV pulsation was a venous triphasic wave composed of four wave patterns (A-, S-, V-, and D-waves) and that the decrease in pulsation was equivalent to the A- and V-waves, which are caused by elevation in the right atrial pressure [7]. The newly defined ICV pulsation index (ICVPI = ICV minimum/maximum velocity) was used to examine ICV pulsation severity [4]. A low ICVPI value indicates that ICV pulsation augmentation and prolonged low ICVPI values are the risk factors for IVH [4].

Herein, we report that augmented ICV pulsation in two premature infants with hemodynamically significant patent ductus arteriosus (hs-PDA) was improved after indomethacin administration.

## Case presentation

Case 1

**Figure 1:**
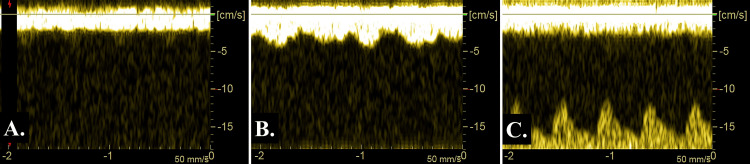
The blood flow pattern of the internal cerebral vein (ICV) in case 1. A) At 25 h after birth, the flow pattern of the ICV was continuously flat (ICV pulsation index (ICVPI) = 1.00); B) At 52 h after birth, the ICV flow pattern demonstrated an augmented pulsatile pattern, with hemodynamically significant patent ductus arteriosus (ICVPI = 0.55); C) After the ductus arteriosus was closed (at 73 h after birth), ICV pulsation improved, adopting a continuously flat pattern (ICVPI = 1.00). * Blood flow in the lower part of the image in Figure 1C indicates the coincidentally and simultaneously obtained pulsatile flow of the cerebral artery.

A female premature infant weighing 999 g was born via cesarean section due to aggravation of her mother’s gestational hypertension at 29 weeks of gestation, with 1- and 5-min Apgar scores of 2 and 6, respectively. The patient was intubated because of respiratory distress syndrome. At 25 h, echocardiography revealed PDA, and the diastolic flow velocity of the left pulmonary artery (DFLPA), for which a value of > 20 cm/s is used as a marker of increased pulmonary blood flow in an hs-PDA [8], was 15 cm/s. At that time, the patient’s ICV blood flow pattern was continuously flat (ICVPI = 1.00) (Figure 1A), and the serum brain natriuretic peptide (BNP) level was 141 pg/ml. After 52 h, the hs-PDA (DFLPA = 35 cm/s) was observed. ICV pulsation was augmented (ICVPI = 0.55) (Figure 1B), with an elevated serum BNP level (1477 pg/ml). Therefore, we administered indomethacin (0.1mg/kg). At 73 h, the PDA closed, and serum BNP levels decreased to 88 pg/ml. The ICV pulsation improved, and its flow pattern became continuously flat (ICVPI = 1.00) (Figure 1C). Thereafter, the infant’s ICV blood flow pattern remained flat, and the ductus arteriosus never re-opened. The infant was discharged from our hospital at 4 months of age without IVH or any other complications. Table 1 presents the transitions of ICVPI, PDA, DFLPA, and BNP levels.

**Table 1:** The chronological change in the internal cerebral vein pulsation index, ductus arteriosus, the diastolic flow of the left pulmonary artery, and serum value of the brain natriuretic peptide in case 1. ICVPI: internal cerebral vein pulsation index; PDA: patent ductus arteriosus; DFLPA: diastolic flow of the left pulmonary artery; BNP: brain natriuretic peptide *Indomethacin administration for hemodynamically symptomatic PDA.

Time after birth (hours)	25	52	73	Day 7
ICVPI	1.00	0.55	1.00	1.00
PDA	(+)	(+)*	(-)	(-)
DFLPA (cm/s)	15	35	6	0
BNP (pg/ml)	141	1477	88	39

Case 2

**Figure 2:**
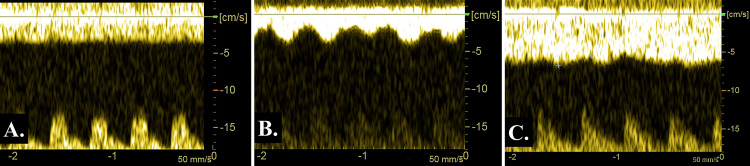
The blood flow pattern of the internal cerebral vein (ICV) in case 2. A) At 15 h after birth, ICV blood flow demonstrated a continuously flat pattern (ICV pulsation index (ICVPI) = 1.00); B) At 39 h after birth, ICV blood flow strongly pulsated with hemodynamically significant patent ductus arteriosus (ICVPI = 0.48); C) After the narrowing of the ductus arteriosus (at 63 h after birth), the ICV pulsation improved and exhibited a mild pulsatile pattern (ICVPI = 0.91). *Blood flow in the lower part of the images in Figures 2A and 2C indicates the coincidentally and simultaneously obtained pulsatile flow of the cerebral artery.

A female premature infant weighing 1693 g was born via cesarean section due to threatened premature delivery and the breech position at 31 weeks of gestation, with 1- and 5-min Apgar scores of 5 and 8, respectively. The infant was intubated because of respiratory distress syndrome. At 15 h, PDA was confirmed along with an 18 cm/s DFLPA. The ICV blood flow pattern was continuously flat (ICVPI = 1.00) (Figure 2A) and the serum BNP level was 209 pg/ml. At 39 h, PDA was exacerbated and DFLPA was increased (35 cm/s) with 2521 pg/ml serum BNP. The ICV pulsation was augmented (ICVPI = 0.48) (Figure 2B). After indomethacin administration (0.1 mg/kg) for hs-PDA, the serum BNP level decreased (181 pg/ml) with narrowing of the ductus arteriosus (DFLPA = 15 cm/s) at 63 h. ICV pulsation decreased and its flow pattern became mildly pulsatile (ICVPI = 0.91) (Figure 2C). Subsequently, the infant did not exhibit severe ICV pulsation or hs-PDA. The infant was discharged from the hospital at 2 months of age without IVH or any other complications. Table 2 presents the transitions of ICVPI, PDA, DFLPA, and BNP levels.

**Table 2:** The chronological change in the internal cerebral vein pulsation index, ductus arteriosus, the diastolic flow of the left pulmonary artery, and serum value of the brain natriuretic peptide in case 2. ICVPI: internal cerebral vein pulsation index; PDA: patent ductus arteriosus; DFLPA: diastolic flow of the left pulmonary artery; BNP: brain natriuretic peptide * Indomethacin administration for hemodynamically symptomatic PDA.

Time after birth (hours)	15	39	63	Day 7
ICVPI	1.00	0.48	0.91	1.00
PDA	(+)	(+)*	(+)	(+)
DFLPA (cm/s)	18	35	15	12
BNP (pg/ml)	209	2521	181	45

## Discussion

In both of these two cases, ICV pulsation was augmented when the PDA became hemodynamically symptomatic along with BNP elevation, while the pulsation decreased when the PDA improved along with BNP reduction. These findings have several implications. First, the finding that hs-PDA increases ICV pulsation may indicate that hs-PDA increases the central venous pressure. To the best of our knowledge, there have been no reports of hs-PDA causing an increased right heart load. Second, indomethacin treatment was effective in reducing augmented ICV pulsation derived from heart failure due to hs-PDA. Thus, treatment for heart failure might improve augmented ICV pulsation, which is related to premature IVH development.

This report revealed that hs-PDA might increase the central venous pressure, which is equivalent to the right atrial pressure. Generally, hs-PDA with excess left-to-right ductal shunting causes left-sided cardiac dysfunction and consecutive venous pulmonary hypertension [9]. Furthermore, pulmonary hypertension results in elevated RV end-diastolic pressure, which is approximated to the right atrial pressure in cases lacking tricuspid stenosis [10,11]. In situations of elevated right atrial pressure, the expansion of the venous wall causes a decrease in vessel distensibility and leads to the distal transmission of venous pulsation [5]. An increase in the right atrial pressure possibly augments ICV pulsation [7]; therefore, excessive pulmonary blood flow due to hs-PDA may be a potential underlying factor of increasing ICV pulsation. Moreover, Ikeda et al. reported that DFLPA increased with pulsatile enhancement in the ICV [12].

Herein, ICVPI values increased or decreased along with serum BNP values, indicating that the two values may be positively correlated. BNP is secreted under volume and pressure overload and is a useful heart failure biomarker [13]. Additionally, right atrial pressure elevation, which augments venous pulsation, contributes to BNP secretion [5,14]. Therefore, pathologies other than hs-PDA that exacerbate right atrial pressure may also intensify ICV pulsation. Moreover, Catalano et al. reported that portal vein pulsation increased with heart failure progression [15].

Based on the aforementioned hypothesis, treatment for heart failure, i.e. load-reduction therapy, might improve augmented ICV pulsation, which is associated with premature IVH development. Singh et al. reported that strong portal vein pulsation substantially improved after the resolution of venous congestion [16]. Additionally, pulsations of the common femoral and portal veins were augmented during heart failure enhancement, while pulsations were reduced after load-reduction therapy [17]. As mentioned above, increased right atrial pressure might cause ICV pulsation enhancement; therefore, load-reduction therapy to decrease right atrial pressure may be effective at preventing premature IVH. In the cases presented herein, closing or narrowing the ductus arteriosus with indomethacin administration effectively improved the increased ICV pulsation by reducing the volume load.

The development of IVH is influenced by multifactorial and heterogeneous factors, including the intrinsic vulnerability of the germinal matrix vasculature. One of these factors is the fluctuation in cerebral blood flow, which can be caused by low Apgar scores, severe respiratory distress syndrome, pneumothorax, hypoxia, hypercapnia, infection, and other conditions. Prenatal glucocorticoids have become the leading intervention for the prevention of IVH, primarily due to their ability to stabilize the germinal matrix's microvasculature and reduce disruptions in cerebral blood flow. Postnatal administration of indomethacin has also been shown to contribute to the prevention of IVH in several clinical studies. However, it is not recommended as a routine method for IVH prevention, as it does not improve neurological outcomes [18]. Our report suggests that indomethacin may reduce the incidence of IVH by decreasing right atrial pressure and improving disturbances in cerebral blood flow.

Augmented venous pulsation is a risk factor for cerebral disorders. Benkreira et al. showed that a significant association exists between the presence of portal pulsatility and delirium [19]. Portal pulsatility is an ultrasound sign of venous congestion, and this cerebral dysfunction might be caused by cerebral venous congestion [20]. Cerebral dysfunction might also occur when the ICV pulsation is augmented; however, this was not confirmed in our report. Further investigation is necessary, such as measuring brain waves during periods of increased ICV pulsation.

This report has some limitations. First, we speculated that ICV pulsation improvement would have been caused by a decrease in the right atrial pressure due to PDA relief; however, we did not measure this pressure directly. However, as both patients were small newborns and their condition was unstable, it was not practical to measure their right atrial pressure. Second, this report is a study of only two cases, therefore, the number of cases is insufficient to conclusively state that reduced load therapy improves ICV pulsation. Further studies enrolling a larger number of participants involving the direct measurement of right atrial pressure are needed to validate our conclusions.

## Conclusions

Overall, in both of the patients presented, we demonstrated that hs-PDA resolution following indomethacin administration improved moderate-to-severe ICV pulsation. This phenomenon is thought to be caused by an improvement in elevated right atrial pressure due to the relief of hs-PDA by indomethacin. Therefore, this experience suggests that load-reduction therapy may be effective in cases of augmented ICV pulsation. Additionally, load-reduction therapy might prevent the development of IVH in premature infants, because the continuation of augmented ICV pulsation is a predictor for developing IVH in premature infants. However, this report is based on a small number of cases; as such, further investigation is needed.
